# Mechanistically resolved prediction of compound hepatotoxicity using primary human liver spheroids—Application to recent real-world cases

**DOI:** 10.1016/j.dmd.2026.100346

**Published:** 2026-06-22

**Authors:** Yufeng Li, Hongda Sheng, Tingting Zhou, Chen Xing, Miriam Kukasch, Siyuan Shao, Sonia Youhanna, Sabine U. Vorrink, Yingxin Liang, Anne T. Nies, Julia Dressler, Charlotte Kölz, Jinhye Ryu, Nayere Taebnia, Dominik Jacob, Caroline Hauser, Carl Petersson, Kathrin Klein, Oliver Burk, Boxu Shi, Qiaojun He, Volker M. Lauschke

**Affiliations:** 1 College of Pharmaceutical Sciences, Zhejiang University, Hangzhou, China; 2 National Key Laboratory of Chinese Medicine Modernization, Innovation Center of Yangtze River Delta, Zhejiang University, Jiaxing, China; 3 Shanghai Hepo Biotechnology Co, Ltd, Shanghai, China; 4 Dr Margarete Fischer-Bosch Institute of Clinical Pharmacology, Stuttgart, Germany; 5 University of Tübingen, Tübingen, Germany; 6 Department of Physiology and Pharmacology and Center for Molecular Medicine, Karolinska Institutet and University Hospital, Stockholm, Sweden; 7 HepaPredict AB, Stockholm, Sweden; 8 Department of Drug Metabolism and Pharmacokinetics (DMPK), the Healthcare Business of Merck KGaA, Darmstadt, Germany; 9 The Chinese University of Hong Kong, Shenzhen, China; 10 Department of Pharmacy, the Second Xiangya Hospital, Central South University, Changsha, China

**Keywords:** 3D spheroids, Drug-induced liver injury, Mitochondrial toxicity, Translational safety, Retrospective validation

## Abstract

Drug-induced liver injury remains a major cause of clinical trial attrition and postmarketing drug withdrawals, reflecting persistent translational gaps in predictive preclinical models of compound hepatotoxicity. Here, we applied a well-characterized 3-dimensional primary human liver spheroid platform to interrogate diverse mechanisms of hepatotoxicity and benchmark performance against recent real-world clinical cases. After multicenter technology transfer, spheroids maintained stable hepatic functionality, including expression of metabolic enzymes and transporters, albumin secretion, and long-term CYP activity. Importantly, repeated-dose exposure to established hepatotoxins demonstrated robust, concentration-dependent toxicity with excellent interexperimental (R^2^ = 0.98) and interdonor (R^2^ = 0.89) reproducibility. To resolve toxicity mechanisms, we implemented Seahorse extracellular flux analysis in liver spheroids, which enabled sensitive detection of early mitochondrial dysfunction, allowing mechanistic discrimination between mitochondrial and nonmitochondrial toxins. Cholestatic liability was identified using bile acid coexposure with accurate classification of chlorpromazine and bosentan and confirmation of bile salt export pump downregulation. Importantly, the model also recapitulated hepatotoxicity signals observed in recent clinical development, including for Bruton’s tyrosine kinase inhibitors (tolebrutinib and evobrutinib), oral glucagon-like peptide-1 receptor agonists (danuglipron vs orforglipron), synergistic toxicity on azelaprag-tirzepatide coexposure and enhanced sensitivity to the IL-17A inhibitor LY3509754 on coculture with nonparenchymal liver cells. Collectively, we present new assays for the mechanistic resolution of hepatotoxicity and provide further evidence that 3-dimensional human liver spheroids enable reproducible, mechanistically informative, and clinically aligned assessment of drug-induced liver injury across multiple modalities, which supports their application for preclinical de-risking of drug candidates.

**Significance Statement:**

This study shows that primary human liver spheroids provide a reproducible and mechanistically informative platform for preclinical assessments of drug metabolism and liver toxicity. The model maintains stable CYP expression and metabolic activity, captures the impact of nonparenchymal cells on hepatic clearance, and detects mitochondrial, cholestatic, and interaction-driven toxicity, including recent real-world clinical cases. These findings support 3-dimensional human liver spheroids as translational new approach methodologies for integrating metabolism and toxicity in drug development.

## 1 Introduction

Drug-induced liver injury (DILI) remains one of the most challenging adverse drug reactions in drug development.^1^^,^^2^ It represents a leading cause of acute liver failure in Western countries and is among the most frequent reasons for clinical trial termination, regulatory restriction, and postmarketing withdrawal of approved medicines.^3^^,^^4^ Despite extensive preclinical safety testing, hepatotoxic liabilities remain problematic in clinical development, underscoring persistent gaps in translational predictivity.^5^ The mechanistic diversity of DILI, which can encompass direct hepatocellular toxicity, mitochondrial dysfunction, oxidative stress, bile acid transport inhibition, immune-mediated injury, and complex drug-drug interactions caused by parent compounds or their metabolites, poses substantial challenges for conventional models used for preclinical hepatotoxicity assessments.^6^^,^^7^

Primary human hepatocytes (PHH) are widely regarded as the gold standard for in vitro liver research; however, their utility in traditional 2D monolayer cultures is limited by rapid dedifferentiation and loss of metabolic competence that can occur within hours.^8^ Liver 3D spheroid cultures have emerged as a promising alternative that enables long-term maintenance of hepatic phenotypes and stable expression of drug metabolizing enzymes and drug transporters for weeks to months.^9^, ^10^, ^11^, ^12^ By preserving cell-cell contacts and an organotypic microenvironment, spheroids enhance the detection of clinically relevant hepatotoxins.^13^, ^14^, ^15^, ^16^ Although primary human liver spheroids have demonstrated clear utility in detecting intrinsic hepatotoxicity and cholestatic liability,^17^ comprehensive mechanistic interrogation across distinct DILI pathways and rigorous benchmarking against contemporary clinical hepatotoxicity cases have thus far only been partially addressed.

The therapeutic landscape is rapidly evolving with multiple novel small-molecule modalities encountering unexpected hepatotoxicity signals during clinical development. For instance, Bruton's tyrosine kinase (BTK) inhibitors, such as tolebrutinib and evobrutinib, have triggered liver enzyme elevations in multiple sclerosis trials, resulting in US Food and Drug Administration (FDA) partial clinical holds and enhanced monitoring requirements.^18^ More recently, the FDA issued a complete response letter rejecting tolebrutinib approval for nonrelapsing secondary progressive multiple sclerosis, citing a serious and unusually high risk of severe DILI, which outweighed potential benefits. Similarly, development of the orally available glucagon-like peptide-1 (GLP-1) agonists lotiglipron and danuglipron was halted due to transaminase elevations and cases of suspected DILI.^19^^,^^20^ These high-profile cases highlight the urgent need for human preclinical systems capable of predicting DILI risk across mechanisms and to indicate safety margins relative to clinical exposure.

In the present study, we applied a well-characterized 3D PHH spheroid platform in a multicenter setting to (1) reestablish functional stability and reproducibility after technology transfer, (2) evaluate the contribution of nonparenchymal cell (NPC) coculture to metabolic and toxicological phenotypes, (3) mechanistically deconvolute mitochondrial toxicity by establishing Seahorse real-time metabolic flux assays for human liver spheroids, and (4) retrospectively assess hepatotoxic liabilities of both benchmark compounds and recent real-world clinical cases. By systematically linking exposure margins, mechanistic signatures, and clinical outcomes, we provide further evidence that primary human liver spheroids constitute a robust and translationally relevant tool for predictive hepatotoxicity assessments.

## 2 Materials and methods

### 2.1 PHH spheroid cultures

Cryopreserved primary human liver cells from 3 donors were commercially obtained from BioIVT or LifeNetHealth. All samples were obtained on informed consent from each donor or the subject’s legally authorized guardian, which was reviewed and approved by the appropriate regulatory authorities in accordance with the U.S. Department of Health and Human Services regulations for the protection of human subjects (45 CFR §46.116 and §46.117) and Good Clinical Practice (ICH E6). Handling of patient-derived cells was approved by the Swedish Ethics Review Authority (Etikprövningsmyndigheten) under permit number 2024-05808-01. Available donor information is shown in Supplemental Table 1. Spheroid culture was conducted as previously described.^21^ Briefly, PHH were seeded either in monoculture or in coculture together with primary human NPCs into 96-well ultralow attachment plates (Corning). For PHH monocultures, 1500 viable cells/well were seeded, whereas cocultures were seeded at a PHH-to-NPC stoichiometry of 5:1 with 1800 viable cells/well in total. Cells were seeded in liver culture medium consisting of Williams’ E medium supplemented with dexamethasone (100 nM), l-glutamine (2 mM), insulin (10 *μ*g/mL), transferrin (5.5 *μ*g/mL), sodium selenite (6.7 ng/mL), penicillin (100 U/mL), streptomycin (100 *μ*g/mL), and 10% FBS. When spheroids were sufficiently compact, FBS was phased out. Compound exposures started on days 7–8 after seeding, and cells were treated with the different compounds in serum-free medium as indicated.

### 2.2 Gene expression profiling

Total RNA was isolated from spheroids at different time points as indicated using the Qiazol lysis reagent (Qiagen) according to the manufacturer’s instructions. RNA concentration and purity were assessed using a NanoDrop spectrophotometer (Thermo Fisher Scientific). RNA was reverse-transcribed into cDNA using the High-Capacity cDNA Reverse Transcription Kit (Applied Biosystems) in a 20 *μ*L reaction volume. Quantitative real-time polymerase chain reaction was performed on a QuantStudio 5 Real-Time PCR System (Applied Biosystems) using PowerUp SYBR Green Master Mix (Applied Biosystems). Primers were designed using Primer-BLAST (Supplemental Table 2).

### 2.3 Determination of CYP enzyme metabolic activity

After 5 days in culture, spheroids were incubated with specific CYP probe substrates (cocktail of phenacetin [50 *μ*M], amodiaquine [10 *μ*M], diclofenac [10 *μ*M], dextromethorphan [5 *μ*M], and midazolam [10 *μ*M]) to assess metabolic activity. The metabolite formation was monitored for up to 72 hours, with all incubations performed in 4 replicates. At the indicated time points, supernatant aliquots were collected and stored frozen at –80 °C. Before analysis, supernatants were transferred into twice the volume of acetonitrile containing 0.5% formic acid and 0.5 *μ*M each of the metabolite-specific internal standards. Samples were centrifuged at 4000*g* for 30 minutes at 4 °C. Supernatants were diluted with 50 mM phosphate buffer before the measurement.

Liquid chromatography-tandem mass spectrometry analysis was conducted using an AB Sciex API 6500+ triple quadrupole mass spectrometer (AB Sciex LLC) coupled with a Waters Acquity I-Class UPLC (Waters Corporation). The instrument was controlled with the software Analyst v1.7 (AB Sciex LLC). Chromatographic separation was achieved on an Acquity UPLC BEH C18, 1.7 *μ*m, 2.1 mm × 50 mm analytical column (186002350; Waters Corporation) at 40 °C. Mobile phase A consisted of 10 mM ammonium formate buffer containing 0.1% (v/v) formic acid, and mobile phase B was acetonitrile. At a flow rate of 0.8 mL/min, the following gradient conditions were used: 0–0.1 minutes 0% B, 0.1–0.8 minutes 0%–100% B, 0.8–1.0 minutes 100% B, and 1.0–1.4 minutes 0% B. Electrospray ionization in positive or negative mode with multiple reaction monitoring was used for detection of the analytes (Table 1). Formation rates of the respective metabolites were calculated and corrected for the cell concentration in the incubation. Calculations were based solely on the linear range of metabolite formation.

**Table 1:** Mass transitions used for determination of metabolic activity

Peak	Enzyme	Polarity	Q1 Mass	Q3 Mass	DP	CE	CXP
			*Da*	*Da*	*Volts*	*Volts*	*Volts*
Acetaminophen	CYP1A2	Pos	152.1	110.1	75	22	11
Acetaminophen-d_4_	CYP1A2	Pos	156.3	114.1	31	23	10
Desethylamodiaquine	CYP2C8	Pos	328.2	282.8	41	30	26
Desethylamodiaquine-(ethyl-d_5_)	CYP2C8	Pos	333.2	282.9	36	44	26
4′-Hydroxydiclofenac	CYP2C9	Neg	310.0	265.9	–20	–18	–14
4′-Hydroxydiclofenac-^13^C_6_	CYP2C9	Neg	315.9	271.9	–35	–18	–17
Dextrorphan	CYP2D6	Pos	258.2	133.3	60	55	12
Dextrorphan-d_3_	CYP2D6	Pos	261.3	157.2	36	60	12
1′-Hydroxymidazolam	CYP3A4	Pos	342.2	297.1	46	52	22
1′-Hydroxymidazolam-d_4_	CYP3A4	Pos	346.0	328.2	46	42	7

CE, collision energy; CXP, collision exit cell potential; DP, declustering potential; Neg, negative; Pos, positive.

### 2.4 Toxicity studies

All test compounds were prepared as stock solutions in DMSO, with the final DMSO concentration in the exposure medium not exceeding 0.3% (v/v). Treatment was initiated on the first day after spheroids had formed, and the medium was refreshed every 2 days thereafter. Samples were collected after 14 days of treatment for ATP quantifications. For cholestasis experiments, cells were treated with or without a pathophysiological bile acid mixture consisting of 20.5 *μ*M cholic acid, 32 *μ*M chenodeoxycholic acid, 24 *μ*M deoxycholic acid, 0.4 *μ*M lithocholic acid, 7 *μ*M ursodeoxycholic acid, and 40 *μ*M glycochenodeoxycholic acid. The cholestatic index (CIx) was calculated as the ratio between the TC_50_ values of treated spheroids with and without bile acids as previously described.^22^

### 2.5 Assessment of viability

Cell viability was assessed using cellular ATP content in individual PHH spheroids as proxy. To this end, ATP levels were measured using the CellTiter-Glo Luminescent Cell Viability Assay (Promega), and results were normalized to vehicle treated controls.

### 2.6 Assessment of albumin secretion

Cell culture medium was collected at different time points, and albumin levels were measured by the human albumin ELISA kit (SEKLH-0005; Solarbio).

### 2.7 Whole-mount staining of liver spheroids

Spheroids were fixed in 4% paraformaldehyde prepared in ice-cold PBS (pH 7.4) at 4 °C for 45 minutes. After fixation, they were permeabilized for 2 hours at room temperature using a permeabilization solution consisting of 0.1% (v/v) Triton X-100, 300 mM glycine, and 20% (v/v) DMSO in PBS. After incubation with primary antibodies against human albumin (ab207327; Abcam, 1:100 dilution), vimentin (5741T; Cell Signaling Technology, 1:100 dilution), or CD68 (76437T; Cell Signaling Technology, 1:400 dilution) overnight at 4 °C, the samples were washed and incubated overnight at 4 °C with the appropriate secondary antibody. Finally, nuclei and F-actin were stained with DAPI (0.5 *μ*g/mL) and phalloidin-Alexa Fluor 488 (1:200 dilution), respectively. All washing steps, as well as antibody and dye dilutions, were performed using a buffer containing 0.1% (w/v) bovine serum albumin, 0.1% (v/v) Triton X-100, and 0.02% (w/v) sodium dodecyl sulfate in PBS.

### 2.8 Confocal imaging

The labeled spheroids were transferred into an 18-well *μ*-Slide (No. 81817; ibidi) and mounted in FUnGI clearing solution as previously described.^23^ After overnight incubation at room temperature, the spheroids were imaged using a Leica Stellaris confocal microscope equipped with an HC PL APO 40×/1.25 GLYC CORR CS2 glycerol-immersion objective. HyD S and HyD X detectors operated in counting mode were used for signal acquisition. The excitation/emission maxima of the fluorophores were as follows: DAPI (nuclei), 358/461 nm; Alexa Fluor 488 (F-actin), 495/520 nm; and Alexa Fluor 555 (albumin or vimentin), 553/569 nm. Images were acquired at a resolution of 1024 × 1024 pixels in the XY plane. Z-stacks were collected with a step size of 1 *μ*m over a depth range of 60–80 *μ*m. Laser intensity Z-compensation was applied to enhance signal detection in deeper optical sections. Three-dimensional reconstruction and visualization were performed using the Leica LAS X 3D Viewer.

### 2.9 Seahorse assay

Mitochondrial activity was profiled using the Seahorse XFe96 Analyzer and FluxPak with the Seahorse XF 3D Mito-Stress Test Assay Kit (Agilent Technologies). The day before the measurement, the test cartridge (Seahorse XFe96 Sensor Cartridge; Agilent Technologies) was hydrated with calibrant according to the manufacturer's instructions. On the day of the measurement, PHH spheroids were carefully placed into the wells of the Seahorse XF 3D capture plate prefilled with 180 *μ*L of Dulbecco's modified Eagle's medium using a wide pipette tip (RT LTS 200 *μ*L BioClean Ultra; Rainin). Care was taken to ensure that the spheroids were positioned in the center of the sensor-active area of the well. For the mitochondrial stress test, 10× stock solutions of oligomycin (40 *μ*M), carbonyl cyanide-p-trifluoromethoxyphenylhydrazone (FCCP; 5 *μ*M), and rotenone/antimycin A (10 *μ*M) were prepared in XF-Dulbecco's modified Eagle's medium (Agilent) and were subsequently filled into the channels A–C of the test cassette according to the manufacturer’s instructions. Calibration and assay runs on the Seahorse XFe96 Analyzer were conducted according to the manufacturer's instructions. The detailed measurement protocol is provided in Supplemental Table 3.

### 2.10 Synergy analyses

The interaction between tirzepatide and azelaprag was quantitatively evaluated using the Zero Interaction Potency (ZIP) model.^24^ To this end, the complete dose-response matrix was analyzed using SynergyFinder 3.0, and ZIP synergy scores were calculated across the entire matrix as well as the most synergistic area. ZIP scores >10 were considered strong synergy, whereas scores of 5–10 indicated moderate synergy and scores <5 indicated no synergy.

### 2.11 Statistical analysis

All statistical analyses were conducted in Prism v.8.0 (GraphPad Inc). Statistical comparisons between 2 groups were conducted using unpaired 2-tailed heteroscedastic *t* tests. No outlier removal was performed. *P* values of <.05 were considered statistically significant.

## 3 Results

### 3.1 Re-establishment of human liver spheroids after multicenter technology transfer

We previously extensively characterized human liver spheroids at the proteomic, transcriptomic, and metabolomic levels over multiple weeks in culture.^21^^,^^25^, ^26^, ^27^ To ensure alignment with these prior data, we first recharacterized key features of primary human liver spheroids after technology transfer to different laboratories. Morphologically, spheroids formed from primary human liver cells in ultralow attachment plates with the expected kinetics, resulting in round spheroidal aggregates of approximately 200 *μ*m in diameter after 6–7 days (Fig. 1A; denoted as day 0). Next, we evaluated the expression of key hepatic markers during spheroid culture. Importantly, levels of all tested CYP isoforms (*CYP1A2*, *CYP2B6*, *CYP2C8*, *CYP2C9*, *CYP2C19*, *CYP2D6*, and *CYP3A4*) remained largely stable over 2 weeks in culture (Fig. 1B). Minor downregulation was observed for *CYP3A4* and *CYP2C8* relative to freshly thawed hepatocytes, but expression stabilized thereafter. In addition, we evaluated albumin secretion over time for 2 weeks as a measure of hepatocyte secretory output. Notably, albumin production remained stable and similar to previous reports,^21^ indicating the maintenance of viability and hepatic functionality without evident dedifferentiation (Fig. 1C).

**Fig. 1:**
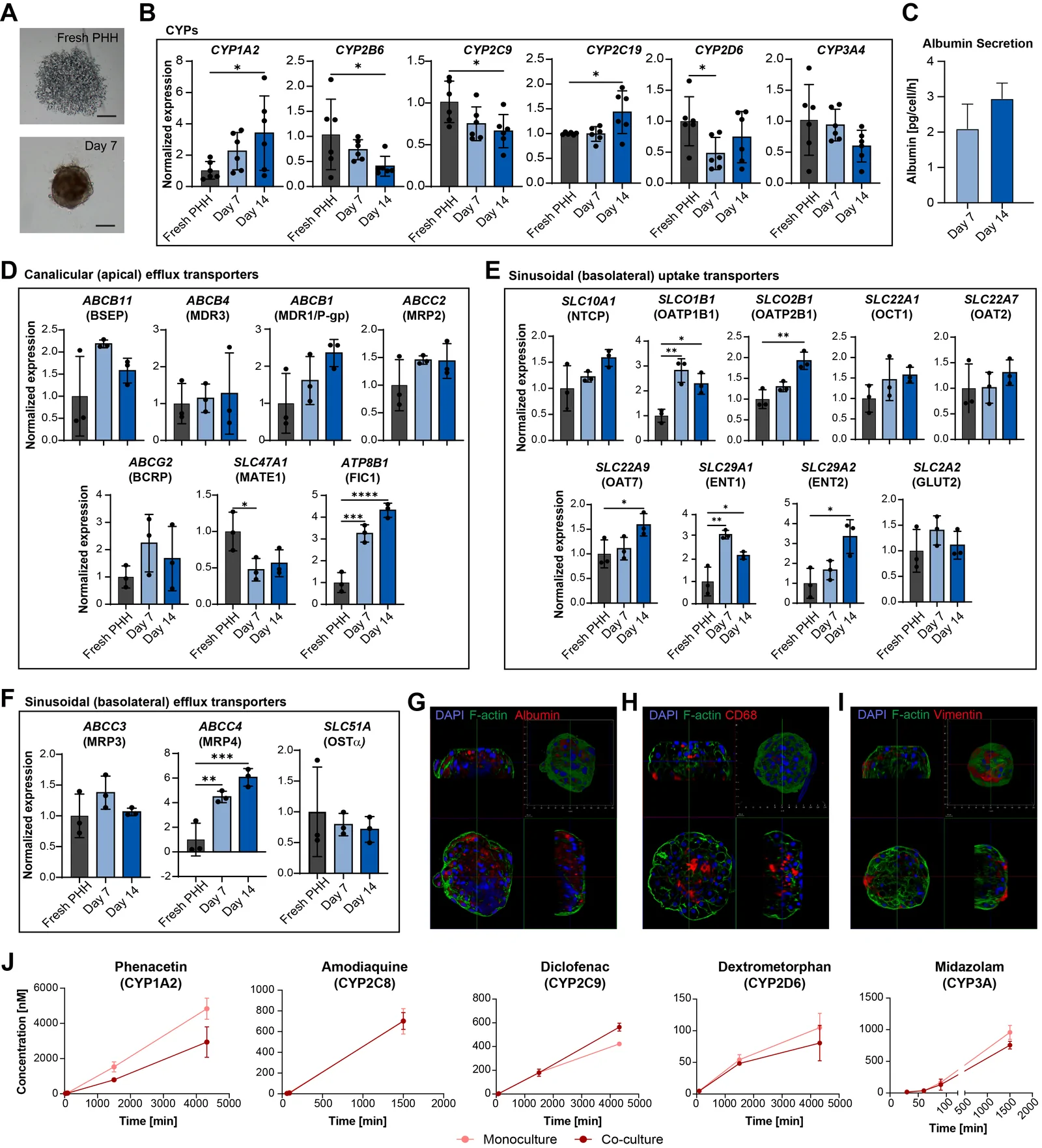
Characterization of primary human liver spheroids after technology transfer. (A) Representative bright-field images of primary human hepatocyte (PHH) spheroids immediately after seeding and at the time of spheroid formation. Scale bar, 100 *μ*m. (B) Relative mRNA expression levels of key pharmacologically important CYP genes are shown normalized to freshly isolated cells. (C**)** Albumin secretion rates expressed in pg per cell per hour during 2 weeks of spheroid culture. (D–F) Relative mRNA expression levels of key apical efflux transporters (D), basolateral uptake transporters (E), and basolateral efflux transporters (F) are shown normalized to freshly isolated cells. (G–I) Confocal immunofluorescence whole-mount staining of coculture spheroids comprising PHH and nonparenchymal liver cells (NPCs). Main view and side view of 3-dimensional reconstructions are shown. Samples were stained with DAPI (nuclei; blue), phalloidin (F-actin; green), and the cell-type-specific markers (red) albumin (G; hepatocytes), CD68 (H; Kupffer cells), and vimentin (I, hepatic stellate cells). (J) Comparison of CYP enzyme activities between PHH monoculture and PHH-NPC co-culture spheroids. Metabolite formation rates measured by mass spectrometry are shown for the indicated substrates. Error bars indicate SD.

Next, we characterized the expression of key canalicular (apical) and sinusoidal (basolateral) transporters in liver spheroids. Among the apical transporters, expression remained stable for bile salt export pump (BSEP), MDR1, MDR3, MRP2, and BCRP for at least 2 weeks in culture, whereas expression of MATE1 was slightly reduced and expression of ATP8B1 was increased (Fig. 1D). Similar results were obtained for basolateral transporters where expression remained stable for all transporters tested with the exception of OATP1B1, OATP2B1, ENT1/2, and MRP4, which slightly increased in expression during the culture period (Fig. 1, E and F). These results show that expression of major drug metabolizing enzymes, as well as apical and basolateral transporters, remains stable in spheroid culture for multiple weeks.

Hepatocytes account for approximately 60% of hepatic cells, whereas the remaining fraction is made up by NPCs, including liver resident macrophages (Kupffer cells) and stellate cells, which contribute critically to the hepatic microenvironment. Although NPCs have been reported to impact the sensitivity to some hepatotoxins in spheroid culture,^28^ their overall impact on drug metabolism and clearance in primary human liver spheroids has not been comprehensively investigated. To enhance pathophysiological relevance, we cocultured PHH with hepatic stellate cells and Kupffer cells isolated from individuals with mild-to-moderate hepatic fibrosis. We first conducted whole-mount immunofluorescence imaging in PHH-NPC coculture spheroids for albumin (marker for hepatocytes), CD68 (Kupffer cells), and vimentin (stellate cells). Three-dimensional reconstruction demonstrates that albumin was broadly expressed throughout the liver spheroid (Fig. 1G). Kupffer cells were predominantly found in the center of the spheroid, whereas stellate cells were enriched in the perimeter (Fig. 1, H and I). These results verify that all surveilled NPC populations were viable and successfully incorporated into cocultured spheroids with distinct localization patterns. Next, we assessed the impact of patient-derived NPCs on hepatic clearance by mass spectrometry. The metabolite formation rate of acetaminophen (CYP1A2) decreased by 39%, whereas the formation of dextrorphan (CYP2D6) and 1OH-midazolam (CYP3A4) decreased by 24% and 22%, respectively (Fig. 1J). In contrast, the formation of 4OH-diclofenac increased by 37%, ruling out that an overall loss in hepatocyte functionality might underlie the observed effects. Importantly, these results align well with clinical data from patients with hepatic cirrhosis in which CYP1A2, CYP2D6, and CYP3A4 activity was decreased whereas CYP2C9 was increased.^29^ Combined, the presented results provide further evidence for the ability of liver spheroids to recapitulate the crosstalk between different hepatic cell populations and their integrated regulation of hepatic CYP metabolism.

### 3.2 Reproducibility of toxicity assessments

Next, we evaluated the hepatotoxicity of a panel of compounds with well-documented clinical evidence of DILI (Table 2).^30^, ^31^, ^32^, ^33^, ^34^, ^35^, ^36^, ^37^, ^38^, ^39^, ^40^, ^41^, ^42^, ^43^ These included drugs whose toxicity mechanism is based on the inhibition of respiratory complexes and/or mitochondrial uncoupling (troglitazone, amiodarone, tolcapone, and diclofenac), formation of reactive metabolites (erlotinib and sorafenib), hepatocellular toxicity due to c-Jun N-terminal kinase activation (efavirenz), and cholestasis (bosentan). Drugs causing mitochondrial impairment exhibited clear concentration-dependent profiles (Fig. 2, A–D). After 14 days of exposure, troglitazone toxicity was detected with a TC_50_ of 22 *μ*M (∼3.5× *C*_max_), whereas TC_50_s for amiodarone, tolcapone, and diclofenac were 12 *μ*M (∼2.4× *C*_max_), 23 *μ*M (∼0.9× *C*_max_), and 48 *μ*M (∼4.7× *C*_max_), respectively. For compounds associated with reactive metabolite formation, both erlotinib and sorafenib exhibited clear hepatotoxicity with TC_50_ values of 4.9 *μ*M (∼1.8× *C*_max_) and 2.8 *μ*M (∼0.3× *C*_max_), respectively (Fig. 2, E and F). Efavirenz exhibited hepatocellular toxicity with a TC_50_ of 11.6 *μ*M (∼1.1× *C*_max_; Fig. 2G), whereas bosentan toxicity was detected at 71.6 *μ*M (∼1× *C*_max_; Fig. 2H).

**Table 2:** Overview of the tested hepatotoxic benchmarking compounds

Drugs	*C*_max_	Class	Toxicity Mechanism	Clinical Features	Reference
Troglitazone	6.4 *μ*M	PPARγ agonist	Mitochondrial toxicity, oxidative stress, and reactive metabolite formation	Severe hepatotoxicity; withdrawn from the market in 2000	30
Amiodarone	0.9–4.7 *μ*M	Primarily K^+^ channel blocker	Phospholipidosis, mitochondrial damage	Significant hepatotoxicity at high doses; mechanisms involve mitochondrial injury and dysfunction, potentially leading to steatosis and inflammation	31
Erlotinib	2.7 *μ*M	EGFR inhibitor	JNK activation, reactive metabolite formation	At least 12 fatal cases reported in the literature, likely due to direct drug toxicity	32
Efavirenz	13 *μ*M	Non-nucleoside reverse transcriptase inhibitor	Sustained activation of JNK. Inhibition of mitochondrial function	1%–8% of patients develop serum transaminase elevations >5× ULN, mostly attributed to immune-mediated reactions	33,34
Sorafenib	10.2–35.9 *μ*M	Raf kinase inhibitor	JNK activation, mitochondrial damage	Up to half of patients experience elevated serum transaminase levels; carries an FDA black box warning; toxicity mechanism may be related to metabolic intermediates	35,36
Tolcapone	21.9 *μ*M	COMT inhibitor	Hepatotoxicity linked to mitochondrial toxicity, oxidative stress, and reactive metabolite formation	Cause serum aminotransferase elevations above 3 times the upper limit of normal in 1%–5% of patients	37,38
Diclofenac	5–10.3 *μ*M	COX-1/2 inhibitor	Inflammation, mitochondrial damage and oxidative stress	The clinical picture is that of jaundice preceded by anorexia, nausea, vomiting, and malaise. Fever and rash occur in 25% of cases, and some cases have immunoallergic features, whereas others resemble chronic hepatitis and have autoimmune features	39,40
Chlorpromazine	0.8 *μ*M	Dopamine D_2_ receptor antagonist	Hepatotoxicity mainly manifests as cholestasis, associated with BSEP inhibition and bile acid transport impairment	Liver test abnormalities have been reported to occur in up to 40% of patients on long-term therapy with chlorpromazine	41
Bosentan	73.2 *μ*M	ETA/ETB antagonist	Hepatotoxicity primarily manifests as cholestasis, linked to BSEP inhibition and bile acid accumulation	Bosentan is associated with elevations in serum aminotransferase levels above 3 times the ULN in 3%–18% of patients	42,43

BSEP, bile salt export pump; COMT, catechol-*O*-methyltransferase; COX-1/2, cyclooxygenase-1/2; EGFR, epidermal growth factor receptor; ETA/ETB, endothelin receptor A/B; FDA, US Food and Drug Administration; JNK, c-Jun N-terminal kinase; PPARγ, peroxisome proliferator-activated receptor gamma; Raf, rapidly accelerated fibrosarcoma protein; ULN, upper limit of normal.

**Fig. 2:**
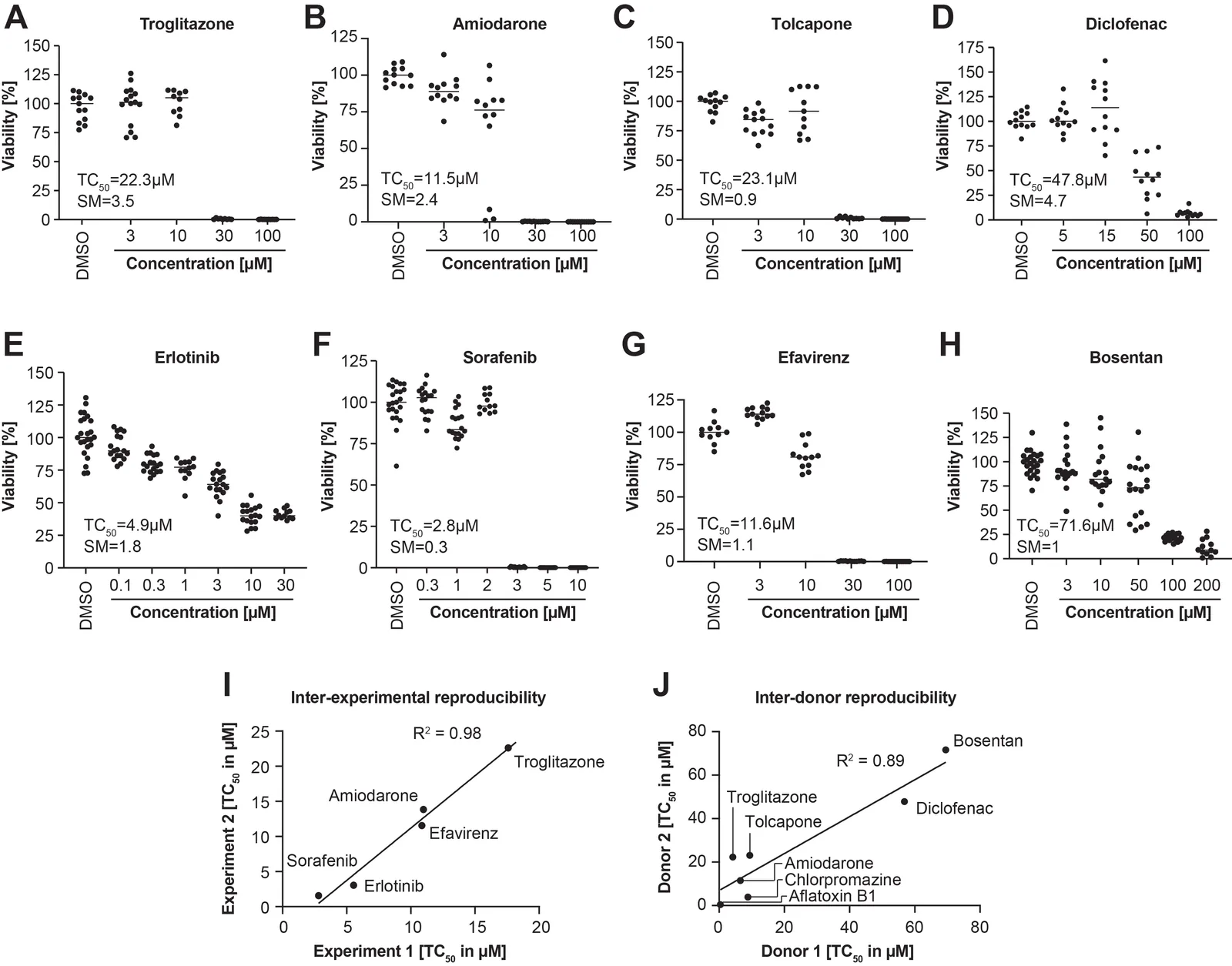
Hepatotoxicity evaluation of known hepatotoxic compounds in primary human hepatocyte spheroids. (A–H**)** Toxicity profiles for the prototypic hepatotoxins troglitazone (A), amiodarone (B), tolcapone (C), diclofenac (D), erlotinib (E), sorafenib (F), efavirenz (G), and bosentan (H) in liver spheroids after 14 days of repeated exposure. Viability was assessed by measuring total cellular ATP content and is expressed as the percentage of vehicle-treated controls. TC_50_ values are indicated for each compound. (I and J) Correlation analysis of TC_50_ values between independent replicate experiments in the same (I) or in different donors (J). Linear regression shows excellent reproducibility for intra- and interdonor variability with R^2^ values of 0.98 and 0.89, respectively. SM, safety margin.

Toxicity of 5 of these 8 compounds was replicated in an independent set of experiments. Importantly, the obtained TC_50_ values were in excellent agreement between experimental replicates (R^2^ = 0.98; Fig. 2I). Furthermore, we compared results with our historical toxicity data for the same culture conditions but generated using different donors.^21^^,^^25^ These comparisons showed that sensitivity is highly similar with a coefficient of determination of 0.89 (Fig. 2J). These results demonstrate the high reproducibility of the spheroid assay for in vitro hepatotoxicity assessments across experimental replicates and donors, which constitutes one of the main prerequisites for assay standardization. Notably though, interdonor differences might be impacted by genetic variability in drug metabolizing enzymes^44^ and for compounds with known pharmacogenetic associations with DILI risk, such as bosentan,^45^ variability between experiments that use hepatocytes from donors with different CYP genotypes might be larger.

### 3.3 Mechanistic deconvolution of mitochondrial and cholestatic toxicity

To measure mitochondrial activity, the Seahorse extracellular flux assay can provide functional measurements of cellular bioenergetics, such as basal respiration, ATP production, maximal respiratory capacity, spare respiratory capacity, and proton leakage by quantifying the oxygen consumption rate in live cells. This assay has been successfully used for the profiling of mitochondrial activity in 2D cultured PHH^46^^,^^47^ as well as in cancer cell spheroids.^48^^,^^49^ However, profiling of primary human liver spheroids has not been described. Because of this gap, we first established a Seahorse protocol for the assessment of primary human liver spheroids. To this end, we compared 2 different setups. In the first, 4 spheroids with 1500 cells each (total 6000 cells) were positioned in the sensor-active area of the Seahorse cell culture plate, and the results were compared to a setup in which 3 spheroids with 3000 cells each (total 9000 cells) were pooled per well. The results showed that a higher initial cell count of 9000 cells per measurement yields higher oxygen consumption rate values, thus allowing for a more reliable determination of changes in mitochondrial activity parameters (Supplemental Fig. 1). Furthermore, the placement of 3 large spheroids in the sensor area of the Seahorse cell culture plate was more precise and contributed to more reliable measurements.

We first evaluated the impact of diclofenac on mitochondrial activity. Diclofenac exhibited a clear time-dependent profile in liver spheroids (Fig. 3A). Importantly, when Seahorse measurements were conducted after exposure, basal respiration and ATP production was drastically reduced already after 24 hours for diclofenac concentrations as low as 10 *μ*M, thus foreshadowing the later drop in viability observed at these concentrations when using total cellular ATP as a proxy (Fig. 3, B–D). In contrast, time-dependent toxicity was weaker in isogenic 2D cultures (Fig. 3E); furthermore, alterations in mitochondrial activity were substantially lower in magnitude and could only be detected at higher concentrations (Fig. 3F). Combined, these results suggest that diclofenac toxicity in human liver spheroids manifests as mitochondrial dysfunction, which precedes the drop in cellular ATP that is typically used as toxicity endpoint. The Seahorse assay thus constitutes a more sensitive method for identifying diclofenac toxicity in liver spheroids, whereas sensitivity is not or only marginally increased in conventional 2D cultures.

**Fig. 3:**
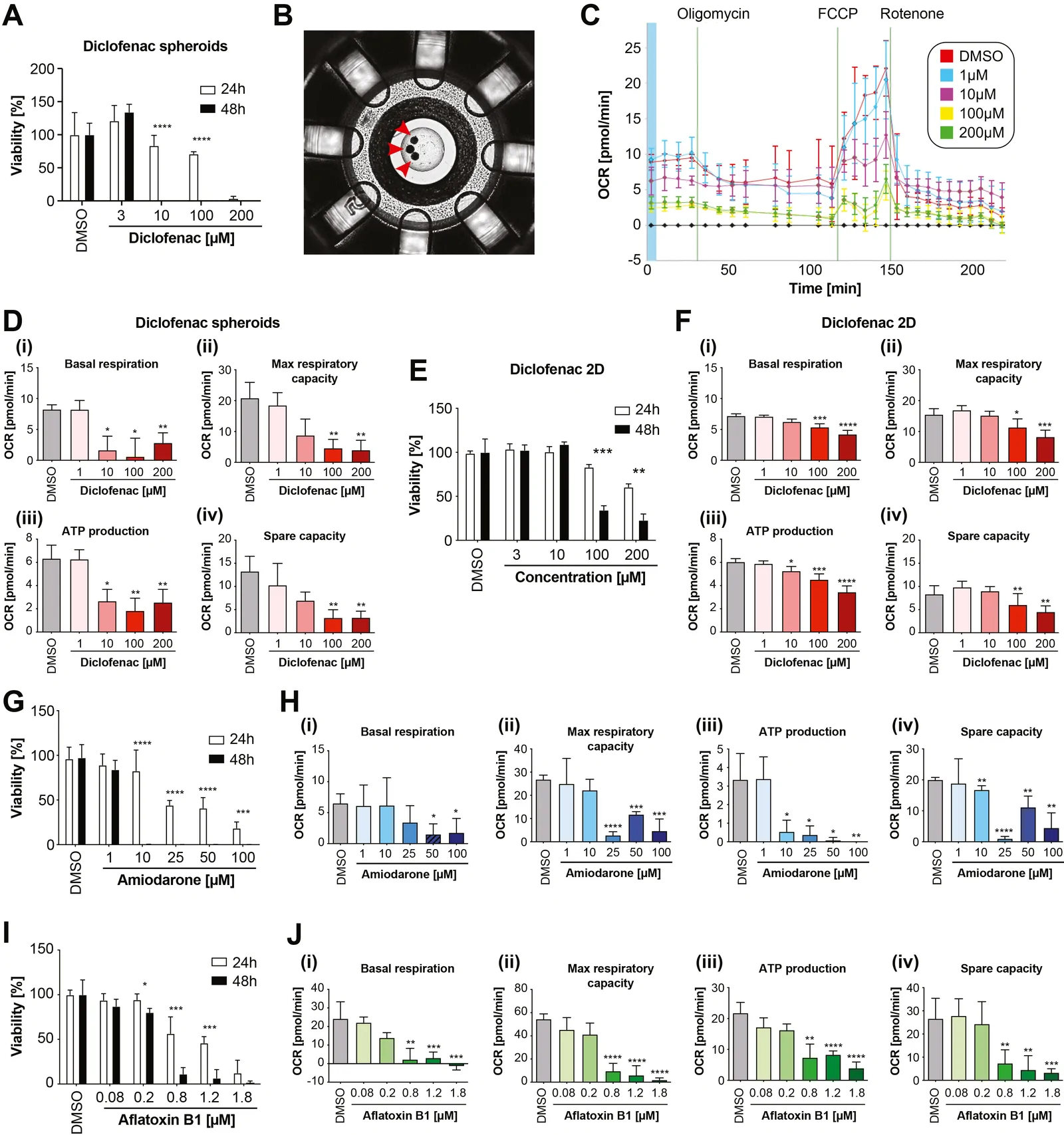
Mitochondrial toxicity assays in liver spheroids using Seahorse extracellular flux analysis. (A) Time- and concentration-dependent effects of diclofenac on PHH spheroid viability after 24 and 48 hours of exposure. Viability was assessed by total cellular ATP content and expressed as the percentage of vehicle-treated controls (mean ± SD, *n* = 3). (B) Representative bright-field image of a Seahorse XF96 well containing 3 pooled liver spheroids. (C) Oxygen consumption rate (OCR) traces in spheroids after exposure to diclofenac or DMSO for 24 hours, measured using the Seahorse XF Spheroid Mito Stress Test. The vertical lines indicate sequential injection of oligomycin, FCCP and rotenone/antimycin A. (D) Quantification of basal respiration (i), maximal respiratory capacity (ii), ATP production (iii), and spare respiratory capacity (iv) in diclofenac-treated spheroids. (E) Time- and concentration-dependent viability of isogenic 2D-cultured PHH exposed to diclofenac (24 hours and 48 hours). (F) Quantification of basal respiration (i), maximal respiratory capacity (ii), ATP production (iii), and spare respiratory capacity (iv) in 2D PHH cultures treated with diclofenac. (G–J) Liver spheroids treated with amiodarone (G–H) and aflatoxin B1 (I and J). Viability of spheroids is shown after exposure to amiodarone (G) and aflatoxin B1 (I) for 24 hours and 48 hours. Quantification of basal respiration (i), maximal respiratory capacity (ii), ATP production (iii), and spare respiratory capacity (iv) is presented for amiodarone- (H) and aflatoxin B1–treated spheroids (J) after 24 hours. Note that drops in viability after 24 hours are foreshadowed for the mitochondrial toxins diclofenac and amiodarone but not for the genotoxic agent aflatoxin B1. ∗*P* < .05; ∗∗*P* < .01; ∗∗∗*P* < .001; ∗∗∗∗*P* < .0001. Error bars indicate SD. 2D, 2-dimensional; FCCP, carbonyl cyanide p-trifluoromethoxyphenylhydrazone; PHH, primary human hepatocyte.

To evaluate Seahorse specificity, we conducted similar evaluations using another drug with known mitochondrial toxicity, amiodarone, as well as aflatoxin B1, which causes genotoxicity rather than mitochondrial dysfunction. Similar to diclofenac, amiodarone exposure resulted in massive drops in maximal respiratory capacity and ATP production in liver spheroids at concentrations of 10 *μ*M already after 24 hours, preceding drops in cell viability (Fig. 3, G and H). In contrast, aflatoxin B1 exposure caused a reduction in mitochondrial activity only at concentrations in which viability was concomitantly impaired, that is, mitochondrial toxicity did not foreshadow reductions in viability (Fig. 3, I and J). These results thus demonstrate that comparison of the concentration and time profiles of mitochondrial activity with overall viability facilitates the mechanistic deconvolution of liver toxicity and can aid in the identification of mitochondrial toxins.

Cholestatic DILI represents an important subtype often linked to inhibition of bile acid transport. To test whether we could identify cholestatic toxicity, we exposed liver spheroids to the prototypic cholestatic agents chlorpromazine and bosentan for 2 weeks with and without bile acid supplementation. For chlorpromazine, we observed a clear increase in sensitivity on bile acid coexposure (Fig. 4A). Calculation of the CIx yielded a value of 0.43 (Fig. 4B). Similar results were obtained for bosentan, for which TC_50_ values dropped from 58.3 *μ*M to 28.8 *μ*M, corresponding to a CIx of 0.49 (Fig. 4, C and D). Thus, both compounds were successfully classified as cholestatic after the established definition of CIx ≤ 0.8. Bosentan exposure significantly inhibited BSEP (*ABCB11*) and MDR3 (*ABCB4*) expression under both standard and bile acid–supplemented conditions (Fig. 4E). In contrast, other bile acid transporters such as *SLC51A* (OST*α*), *SLC51B* (OST*β*), *ABCC3* (MRP3), and *ABCC4* (MRP4) were induced on bile acid exposure; however, their expression was not blunted by bosentan (Supplemental Fig. 2). These findings support the hypothesis that repression of BSEP and MDR3 constitutes the principal mechanism underlying bosentan hepatotoxicity.

**Fig. 4:**
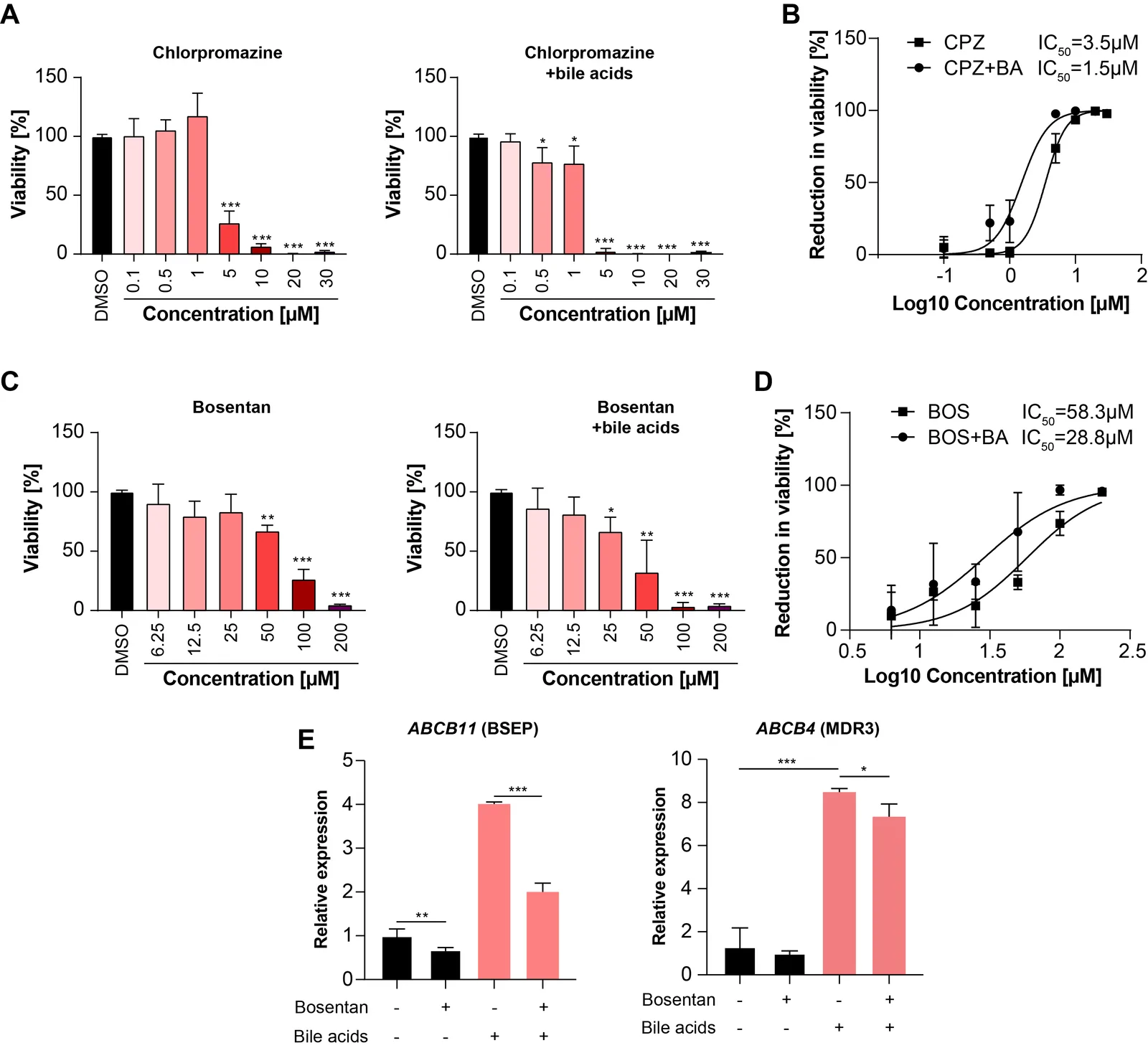
Identification of cholestatic toxicity risk. (A) Concentration-dependent effects of chlorpromazine on liver spheroid viability with or without coexposure to a pathophysiological bile acid mixture for 14 days. (B) The resulting TC_50_ values for chlorpromazine show a significant increase in sensitivity on bile acid exposure. (C) Concentration-dependent effects of bosentan on liver spheroid viability with or without coexposure to a pathophysiological bile acid mixture for 14 days. (D) The resulting TC_50_ values for bosentan show a >2-fold increase in sensitivity on bile acid exposure, suggesting cholestatic injury. (E) Relative mRNA expression of BSEP (*ABCB11*) and MDR3 (*ABCB4*) in liver spheroids treated with bile acids and/or 30 *μ*M bosentan. ∗*P* < .05; ∗∗*P* < .01; ∗∗∗*P* < .001. Error bars indicate SD. BA, bile acid; BOS, bosentan; CPZ, chlorpromazine.

### 3.4 Evaluation of recent real-world cases of drug-induced hepatotoxicity using liver spheroids

We next used the established spheroid models to assess the hepatotoxic potential of compounds from 2 emerging therapeutic classes with recent documented clinical liver safety concerns, BTK inhibitors and GLP-1 receptor agonists. Compounds were administered repeatedly over 14 days at concentrations scaled to multiples of their reported clinical *C*_max_ values (0.58 *μ*M for evobrutinib and 0.12 *μ*M for tolebrutinib). For BTK inhibitors, tolebrutinib produced marked cytotoxicity already starting at 6× *C*_max_ (Fig. 5A), in line with clinical reports of serious DILI that led to FDA rejection of its marketing application due to unacceptable hepatotoxicity risk. Similarly, evobrutinib induced significant toxicity at concentrations below 1× *C*_max_ (Fig. 5B), consistent with phase 2 observations of transaminase elevations in 26% of patients and toxicogenomic predictions of hepatotoxic potential.^18^

**Fig. 5:**
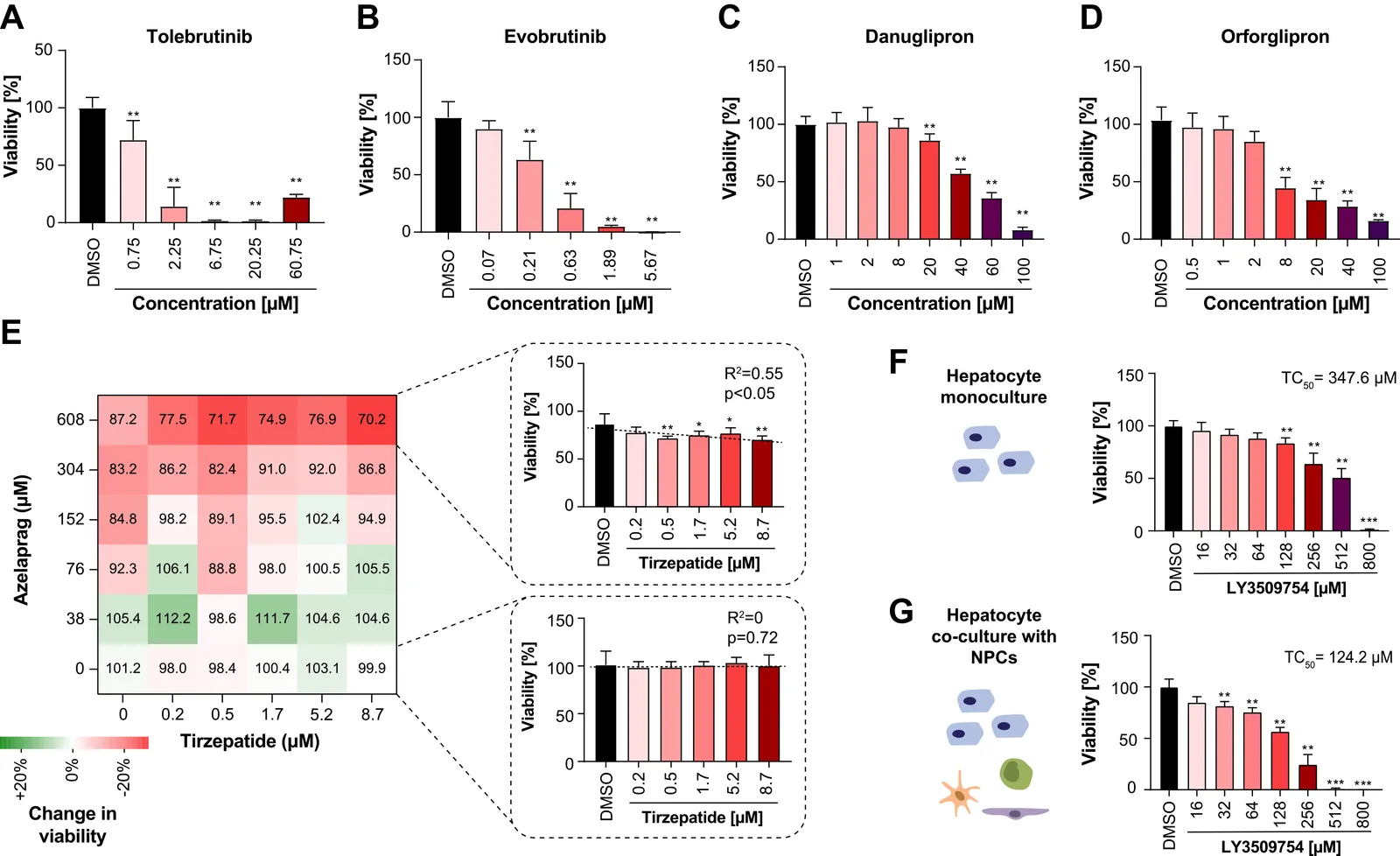
Evaluation of recent real-world DILI cases in liver spheroids. (A–D) Concentration-dependent effects of tolebrutinib (A), evobrutinib (B), danuglipron (C), and orforglipron (D) on PHH spheroid viability. (E) Matrix of exposure combinations for liver spheroids treated with azelaprag and tirzepatide for 14 days (left). Exemplary bar graphs for the highest and the lowest azelaprag concentrations are shown (right). Note the significant decrease of cell viability on azelaprag-tirzepatide coexposure (R^2^ = 0.55), whereas no trend is apparent for tirzepatide alone (R^2^ = 0). (F and G) The toxicity of the IL-17A inhibitor LY3509754 is shown in PHH monoculture spheroids (F) and in PHH-NPC coculture spheroids (G). Note that coculture significantly increases spheroid sensitivity. ∗*P* < .05; ∗∗*P* < .01; ∗∗∗*P* < .001. Error bars indicate SD. DILI, drug-induced liver injury; NPC, nonparenchymal cell; PHH, primary human hepatocyte.

GLP-1R agonists are widely used for the treatment of diabetes, obesity, and cardiometabolic disease.^50^ In recent years, development trends have shifted away from peptidic GLP-1 agonists toward orally available small molecules, such as danuglipron and orforglipron. Although hepatocytes are generally considered to not express functional GLP-1R, danuglipron was discontinued after phase 2 trials due to DILI.^19^ In contrast, no hepatic safety signals were observed for orforglipron.^51^ In liver spheroids, danuglipron elicited significant, dose-dependent cytotoxicity already at 15× of the therapeutic *C*_max_ (Fig. 5C); in contrast, orforglipron elicited notable toxicity only at concentrations >30× *C*_max_ (Fig. 5D). These results suggest a higher safety margin of orforglipron aligning with the corresponding clinical data.

Next, we investigated the synergy of GLP-1R and apelin receptor (APJ) agonists in causing DILI. The combination of the APJ agonist azelaprag with the dual GLP-1R/GIPR agonist tirzepatide was associated with liver injury cases in the STRIDES trial, despite both agents appearing safe as monotherapies.^52^ In agreement with these clinical results, neither monotherapy caused significant toxicity at any of the concentrations tested (Fig. 5E). To test whether liver spheroids might identify the interaction of the 2 drugs, we conducted a matrix titration of azelaprag and tirzepatide. On cotreatment, we observed that tirzepatide amplified azelaprag hepatotoxicity, resulting in significant toxicity at 15× of azelaprag *C*_max_. To further substantiate these results, we conducted formal synergy analyses using SynergyFinder 3.0.^24^ At high concentrations of azelaprag, the synergy score reached +11.33, indicative of a strong synergy, whereas ZIP scores at lower azelaprag concentrations did not show synergy (ZIP scores <5; Supplemental Fig. 3). These results constitute an interesting and clinically relevant case in which 3D human liver spheroids can confirm unexpected synergistic hepatotoxicity that recapitulates the clinical findings.

Lastly, we assessed hepatotoxicity of LY3509754, a small molecule IL-17A inhibitor developed for treatment of psoriasis that caused acute hepatitis in a phase 1 multiple ascending dose trial.^53^ In PHH monoculture spheroids, we observed hepatotoxicity with a TC_50_ of 347 *μ*M; however, this was amplified in PHH-NPC coculture spheroids (TC_50_ = 124 *μ*M). Based on these results, we hypothesize that the liver toxicity is at least in part mediated by nonparenchymal inflammatory signaling, which would be in agreement with the trial’s biopsy results that showed lobular inflammation. Jointly, these findings add to the growing body of literature showing that liver spheroids provide a versatile tool for the identification and mechanistic deconvolution of hepatotoxicity mechanisms during preclinical development.

## 4 Discussion

DILI remains a major impediment to efficient drug development. The persisting clinical safety challenges reflect the complexity and diversity of toxicity mechanisms as well as the limited translational performance of conventional preclinical models. Consequently, predictive systems must not only detect overt cytotoxicity but also enable mechanistic resolution and contextualization relative to clinical exposure. In this study, we evaluated the performance of our 3D human liver spheroid platform across benchmark hepatotoxins and recent real-world clinical cases in a decentralized multicenter setting. Importantly, we found that the molecular phenotypes and sensitivity to prototypic hepatotoxins were very similar between sites with high interexperimental reproducibility after technology transfer (R^2^ = 0.98). For the tested compound set, we also observed overall low variability in sensitivity across donors (R^2^ = 0.89). Previous studies showed substantial 13- to 860-fold differences between donors in their transcriptomic responses to toxicological stimuli.^54^ In contrast, interindividual variability at the level of sensitivity to hepatotoxic drugs was also previously found to be substantially smaller with TC_50_s differing <2-fold across a panel of 6 drugs and 3 donors.^55^ Notably however, these findings do not rule out that certain donors could show drastically different responses to specific hepatotoxic drugs. Reproducibility remains a critical bottleneck for regulatory acceptance of new approach methodologies, and the data presented here thus provide further evidence that toxicity assessments using spheroids cultured in chemically defined media remain stable across experiments, sites, and donors.

Notably, we observed that coculture of PHH with NPCs from patients with metabolic dysfunction-associated steatohepatitis (MASH) reduced the activity of CYP1A2, CYP2D6, and CYP3A4, whereas CYP2C9 metabolism was increased. These results closely match with clinical data from patients with hepatic cirrhosis in which CYP1A2, CYP2D6, and CYP3A4 activities are decreased, whereas CYP2C9 is increased.^29^ Previous studies reported a slight decrease in CYP2D6 in cocultured spheroids, whereas CYP1A2 and CYP3A4 were not significantly impacted.^56^ However, comparison of our results with these findings is complicated by the fact that PHH-to-NPC ratios are not provided, and the reported spheroid activity in this study was >3-fold lower for CYP2C8 and up to 40-fold lower for CYP1A2 and CYP3A4 compared with our previous experiments^57^ as well as to the metabolic activity of spheroids presented here.

Our results furthermore extend the mechanistic capability of liver spheroids by establishing Seahorse-based extracellular flux analysis in intact 3D culture. Mitochondrial dysfunction is recognized as a central initiating event in many forms of DILI.^58^ Importantly, we observed that mitochondrial impairment preceded loss of viability in spheroids treated with the mitochondrial toxins diclofenac and amiodarone, whereas this was not the case when using the genotoxic agent aflatoxin B1. Evaluation of these toxicity dynamics thus allows for early mechanistic discrimination of drugs with mitochondrial and nonmitochondrial mechanisms of toxicity. In contrast, 2D cultures were markedly less sensitive and displayed attenuated and delayed bioenergetic responses, which underlines the importance of using culture models in which metabolic competence is maintained. These findings expand the experimental repertoire for mechanistic toxicity studies beyond cholestasis, inflammation, and toxicogenomics, providing a further functionally informative assay capable of resolving initiating injury events.

Beyond reproducibility analyses and expansion of toxicity assay diversity, an important objective of this work was to expand our retrospective toxicity set^15^ with recently emerging clinical hepatotoxicity signals. Here, we show that the spheroid system recapitulated toxicity profiles of BTK inhibitors, differentiated between oral GLP-1 receptor agonists with distinct clinical safety outcomes and detected synergistic hepatotoxicity of azelaprag-tirzepatide coexposure. Notably, this constitutes only the second toxic drug-drug interaction besides tenofovir-inarigivir^59^ that has been identified in 3D human liver cultures. Furthermore, the results demonstrated enhanced sensitivity to the IL-17A inhibitor LY3509754 in the presence of hepatic immune cells, showcasing that complex liver microtissues can recapitulate immunogenic hepatotoxic risk. Although this configuration does not yet model adaptive immune mechanisms, it represents an important step toward Tier 3 complexity as outlined in recent expert frameworks.^5^ These retrospective results moreover demonstrated that use of liver spheroids would have correctly flagged potential hepatotoxicity at commonly accepted safety margins of 30× of therapeutic *C*_max_. Although not systematic, our results expand the available data for alignment between exposure-scaled safety signals within a metabolically competent 3D human liver system and clinical observations. Notably, these associations include complex cases, such as inflammation-associated injury and injury arising from drug to drug interactions.

Several limitations warrant consideration. First, although exposure margins aligned with clinical outcomes in tested cases, continuous expansion of the test set, also including nonhepatotoxic negative controls will be helpful to refine quantitative predictive thresholds and increase predictive accuracy for prospective use. Second, ATP-based viability remains a sensitive but late endpoint for toxicity. Furthermore, ATP levels are not only changed on cell death but can also be impacted by metabolic modulation and hypertrophy. Thus, ATP quantifications can only serve as a proxy for viability and incorporation of orthogonal readouts such as high-content imaging, cell painting, mitochondrial activity measures, or transcriptomic stress signatures can provide important confirmation and mechanistic resolution. Finally, Although NPC coculture improved inflammatory context, the system does not yet incorporate circulating immune cells or patient-specific genetic susceptibility factors, which could further expand capabilities to detect idiosyncratic toxicity events.

Collectively, our findings support the concept that 3D human liver spheroids in chemically defined conditions represent a robust and mechanistically informative platform for preclinical hepatotoxicity profiling and toxicomechanistic assessments. By enabling reproducible detection of diverse DILI mechanisms, mechanistic discrimination of different injury modalities, and alignment with contemporary clinical safety signals, this platform can enable earlier identification of hepatotoxic liabilities and improved derisking strategies in drug development. Continued integration with multiomics profiling, immune components, and quantitative exposure modeling will further enhance the translational utility and regulatory relevance of liver spheroids for DILI risk assessment.

## Conflict of interest

Volker M. Lauschke is CEO and shareholder of HepaPredict AB, as well as co-founder and shareholder of Shanghai Hepo Biotechnology Ltd. Tingting Zhou is CEO of Shanghai Hepo Biotechnology Ltd. Carl Petersson and Dominik Jacob are employed by Merck Healthcare KGaA, which developed and invented evobrutinib. All other authors declare no conflicts of interest.
