# Supplementary material for: Mechanistically resolved prediction of compound hepatotoxicity using primary human liver spheroids—Application to recent real-world cases
Source: Drug Metab Dispos. 2026 Jun 22;54(8):100346. doi: 10.1016/j.dmd.2026.100346 (PMC13688977; doi:10.1016/j.dmd.2026.100346)

Supplemental Figure 3

0	<5	<5	<5	<5	11.33
0	<5	<5	<5	<5	8.15
0	<5	<5	<5	<5	8.21
0	<5	<5	<5	<5	8.66
0	<5	<5	<5	<5	4.04
0	0	0	0	0	0

ZIP Synergy score

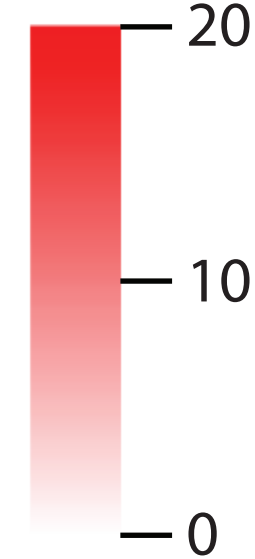

Supplement: Supplementary file 3 [file mmc2.pdf]
