# Supplementary material for: Mechanistically resolved prediction of compound hepatotoxicity using primary human liver spheroids—Application to recent real-world cases
Source: Drug Metab Dispos. 2026 Jun 22;54(8):100346. doi: 10.1016/j.dmd.2026.100346 (PMC13688977; doi:10.1016/j.dmd.2026.100346)
Supplement: Supplementary file 7 [file mmc3.docx]

**Supplemental Table 1. Medical and demographic information of liver cell donors.**

| **Donor** | **Sex** | **Ethnicity** | **Age** | **BMI** | **Cause of death** | **Medications** |
| --- | --- | --- | --- | --- | --- | --- |
| 1 | F | Caucasian | 42 | 26.9 | Anoxia | None |
| 2 | F | Hispanic | 40 | 25.3 | Anoxia/Blunt Injury/Head Trauma | Insulin |
| 3 | F | Hispanic | 45 | 32 | Anoxia | None |
