# Supplementary material for: Mechanistically resolved prediction of compound hepatotoxicity using primary human liver spheroids—Application to recent real-world cases
Source: Drug Metab Dispos. 2026 Jun 22;54(8):100346. doi: 10.1016/j.dmd.2026.100346 (PMC13688977; doi:10.1016/j.dmd.2026.100346)
Supplement: Supplementary file 8 [file mmc4.docx]

**Supplemental Table 2. Sequences of the utilized qPCR primers.**

| **Gene name** | **Forward** | **Reverse** |
| --- | --- | --- |
| *CYP3A4* | TGGAAAAGTGTGGGGCTTTT | AAAGGCCTCCGGTTTGTGAA |
| *CYP2D6* | CCAACGGTCTCTTGGACAAAG | GGGTCGTCGTACTCGAAGC |
| *CYP1A2* | CTGGGCACTTCGACCCTTAC | TCTCATCGCTACTCTCAGGGA |
| *CYP2B6* | GCACTCCTCACAGGACTCTTG | CCCAGGTGTACCGTGAAGAC |
| *CYP2C8* | CATGGATATGAGGCAGTGAAGG | CCATCCCAAAATTCCGCAAGG |
| *CYP2C9* | AAGATTTTGAGCAGCCCCTG | TTGGTTGTGCTTTTCCTTCTCC |
| *CYP2C19* | GAAAAACCAAGGCTTCACCC | AGGGGGTGCTTACAATCCT |
| *GAPDH* | GGAGCGAGATCCCTCCAAAAT | GGCTGTTGTCATACTTCTCATGG |
