# Supplementary material for: Mechanistically resolved prediction of compound hepatotoxicity using primary human liver spheroids—Application to recent real-world cases
Source: Drug Metab Dispos. 2026 Jun 22;54(8):100346. doi: 10.1016/j.dmd.2026.100346 (PMC13688977; doi:10.1016/j.dmd.2026.100346)
Supplement: Supplementary file 9 [file mmc5.docx]

**Supplemental Table 3.** **Protocol for the mitochondrial stress test of liver spheroids in the Seahorse XFe96 Analyzer.**

| **Step** | **Time in min:sec** | **Repeats** |
| --- | --- | --- |
| Baseline | | |
| Mix | 01:00 | 4 |
| Wait | 01:00 |  |
| Measure | 06:00 |  |
| Injection of oligomycin | | |
| Mix | 01:00 | 4 |
| Wait | 01:00 |  |
| Measure | 06:00 |  |
| Wait | 10:00 |  |
| Mix | 01:00 | 2 |
| Wait | 01:00 |  |
| Measure | 06:00 |  |
| Wait | 10:00 |  |
| Mix | 01:00 | 2 |
| Wait | 01:00 |  |
| Measure | 06:00 |  |
| Injection of FCCP | | |
| Mix | 01:00 | 5 |
| Wait | 01:00 |  |
| Measure | 04:00 |  |
| Injection of rotenone/antimycin A | | |
| Mix | 01:00 | 11 |
| Wait | 01:00 |  |
| Measure | 04:00 |  |
