# Supplementary material for: Mechanistically resolved prediction of compound hepatotoxicity using primary human liver spheroids—Application to recent real-world cases
Source: Drug Metab Dispos. 2026 Jun 22;54(8):100346. doi: 10.1016/j.dmd.2026.100346 (PMC13688977; doi:10.1016/j.dmd.2026.100346)
Supplement: Supplementary file 10 [file mmc6.docx]

**Supplementary Material**

Supplemental Tables: 3

Supplemental Figures: 3

**Supplemental Table 1. Medical and demographic information of liver cell donors.**

**Supplemental Table 2. Sequences of the utilized qPCR primers.**

**Supplemental Table 3.** **Protocol for the mitochondrial stress test of liver spheroids in the Seahorse XFe96 Analyzer.**

**Supplemental Figure 1. Spheroid titration for Seahorse extracellular flux analysis. a**, Oxygen consumption rate (OCR) traces measured using the Seahorse XF Spheroid Mito Stress Test. Results are shown for assays in which three spheroids with 1,500 cells (total 4,500 cells; blue) or three spheroids with 3,000 cells (total 9,000 cells; red) were pooled per well. Vertical lines indicate sequential injection of oligomycin, FCCP and rotenone/antimycin A. **b**, Quantification of basal respiration (i), maximal respiratory capacity (ii), ATP production (iii) and spare respiratory capacity (iv) in spheroids of different sizes.

**Supplemental Figure 2. Relative expression of bile acid transporters in liver spheroids treated with bile acids and/or bosentan.** mRNA expression levels of *SLC51A* (OSTα; **a**), *SLC51B* (OSTβ; **b**), *ABCC3* (MRP3; **c**) and *ABCC4* (MRP4; **d**) are shown for spheroids treated with a pathophysiological bile acid cocktail and/or 30 µM bosentan. *p<0.05, **p<0.01, ***p<0.001, ****p<0.0001. Error bars indicate SD.

**Supplemental Figure 3. ZIP synergy scores across the tirzepatide-azelaprag interaction matrix.** Synergy scores were calculated using SynergyFinder 3.0 (see Methods). ZIP synergy scores >10 indicates strong synergy while scores of 5–10 indicate moderate synergy.
